# A Rare Case of Meralgia Paresthetica: Lateral Femoral Cutaneous Nerve Schwannoma Mimicking Appendicitis

**DOI:** 10.7759/cureus.115027

**Published:** 2026-08-23

**Authors:** Snehalakshmi Kavacheri Subramaniam, Sheik Asik Abu Sali, Rajesh Kumar S

**Affiliations:** 1 Surgery, PSG Institute of Medical Sciences and Research, Coimbatore, IND

**Keywords:** benign peripheral nerve sheath tumor, lateral femoral cutaneous nerve (lfcn), meralgia paresthetica, peripheral schwannoma, refractory meralgia paresthetica, retrocecal appendicitis

## Abstract

Meralgia paresthetica (MP) is a mononeuropathy caused by compression of the lateral femoral cutaneous nerve (LFCN), producing pain, numbness, and paresthesia over the anterolateral thigh. While MP is commonly due to benign compressive or metabolic causes, it may rarely result from structural lesions such as LFCN schwannomas. We report a 50-year-old woman with a one-month history of right lower quadrant abdominal pain and anterolateral thigh numbness, followed by cramping pain extending into the right leg. She also described a burning sensation in both soles. Examination revealed right iliac fossa tenderness with a positive psoas sign and sensory loss over the anterolateral thigh. Initially, a diagnosis of retrocecal appendicitis was considered. The straight leg raising test was negative, but leg elevation reproduced pain in the right iliac fossa, and neurodynamic testing (LFCN stretch test) reproduced pain over the lateral thigh. Imaging showed a dumbbell-shaped lesion along the right psoas muscle. Laparoscopy confirmed a multilobulated mass in direct continuity with the LFCN, which was completely excised, and the nerve was preserved. Histopathology confirmed a benign schwannoma with degenerative changes. At one month's follow-up, her abdominal pain had resolved, and thigh numbness had improved, though bilateral sole burning persisted and remained unexplained. This case highlights LFCN schwannoma as a rare structural cause of MP that can mimic chronic appendicitis, and it underscores the need to consider structural causes in refractory or atypical thigh pain with abdominal symptoms. It is also important to use imaging and histopathology for timely diagnosis and surgical management.

## Introduction

Meralgia paresthetica (MP) is a nerve entrapment neuropathy resulting in pain, paresthesia, and sensory loss within the distribution of the lateral femoral cutaneous nerve (LFCN). This condition has been associated with a range of mechanical, metabolic, and idiopathic causes [1,2]. Mechanical causes include compression due to obesity, pregnancy, tight garments, scoliosis, iliacus hematoma, seat belt injury, trauma, and others. Metabolic factors include diabetes mellitus, alcoholism, hypothyroidism, and lead poisoning [3]. The LFCN arises from the L2 and L3 nerve roots, emerges at the lateral border of the psoas major, courses over the iliacus muscle, and passes under the inguinal ligament near the anterior superior iliac spine (ASIS) before dividing into anterior and posterior branches, which provide sensation to the anterolateral and lateral aspects of the thigh, respectively [4,5]. MP more commonly involves the anterior division [5]. We report a case of an uncommon cause of MP, an LFCN schwannoma, in which the patient presented with right iliac fossa pain initially suspected to be chronic appendicitis. She also reported anterolateral thigh numbness. This pointed to a structural lesion rather than ordinary MP or appendicitis. Chronic right iliac fossa pain should prompt a complete neurological exam because it can mimic a neurological condition. In our case, chronic abdominal pain was present due to the size, its proximity to the psoas muscle, and its proximal location relative to the inguinal ligament. Due to the retroperitoneal location, the patient might have presented with abdominal pain. This unusual presentation highlights both an uncommon etiology of MP and its atypical manifestation mimicking other common causes of abdominal pain.

## Case presentation

A 50-year-old woman, a homemaker with no comorbidities and a normal BMI, presented with a one-month history of dull, aching right lower quadrant abdominal pain, aggravated by right leg movement, and numbness over the anterolateral aspect of the right thigh. Both these symptoms started together. Over the following weeks, she additionally developed cramping pain extending into the right leg and a burning sensation in both soles. She had no complaints of fever, vomiting, or bowel and bladder disturbances. There is no history of trauma, use of tight clothing, pregnancy, or prior abdominal or pelvic surgery. She had been taking over-the-counter analgesics and undergoing physical therapy, with no improvement.

On examination, vital signs were stable. The abdomen was soft with localized tenderness in the right iliac fossa; the overlying skin was normal, and no mass was palpable. The psoas sign was positive; the obturator sign and rebound tenderness were absent. This combination of localized right iliac fossa tenderness and a positive psoas sign--both classically associated with appendicitis, particularly a retrocecal appendix irritating the psoas muscle--was the basis for the initial clinical suspicion of chronic retrocecal appendicitis.

Neurological examination of the right lower limb revealed loss of pain and temperature sensation over the anterolateral aspect of the right thigh. The sensory deficit extended from approximately 1.5 cm below the ASIS to 1 cm above the knee, involving the full anterolateral width of the thigh. Touch, vibration, and proprioception were intact in both lower limbs, and tone, power, bulk, and reflexes were normal, with a full range of movement at the hip and knee. Bilateral sole sensation was normal. The straight leg raising test was negative, but pain was reproduced in the right iliac fossa on elevation of the right leg (psoas sign). The pelvic compression test was performed with the patient in the left lateral decubitus position (right side up), with sustained downward compression applied over the right iliac crest for 45 seconds; this did not relieve her symptoms. Neurodynamic testing (LFCN stretch test) was performed with the patient in the left lateral decubitus position and the right hip passively extended and adducted, reproducing her anterolateral thigh pain. The burning sensation in both soles did not correspond to the distribution of any single lesion and was considered clinically consistent with small fiber neuropathy. Further evaluation was deferred, as the patient refused to undergo further studies, which is a limitation of this case. Routine blood investigations were within normal limits (Table 1).

**Table 1:** Laboratory investigations of the patient.

Lab tests	Result	Reference range
Hemoglobin	13 g/dL	12-17 g/dL
WBC count	7.8 × 10³/µL	4.0-11.0 × 10³/µL
Neutrophils	48.3%	40-75%
Lymphocytes	38.9%	20-45%
Monocytes	7.1%	2-10%
Eosinophils	5.2%	1-6%
Basophils	0.5%	0-2%
Platelet count	284,000/µL	150,000-450,000/µL
Serum urea	15 mg/dL	7-20 mg/dL
Serum creatinine	0.65 mg/dL	0.5-1.3 mg/dL
Sodium	138 mEq/L	135-145 mEq/L
Potassium	4.83 mEq/L	3.5-5.0 mEq/L
Chloride	110 mEq/L	98-110 mEq/L
Bicarbonate	22.5 mEq/L	22-29 mEq/L
Ionized calcium	1.13 mmol/L	1.10-1.35 mmol/L
PT INR	0.93	0.8-1.1

**Figure 1:**
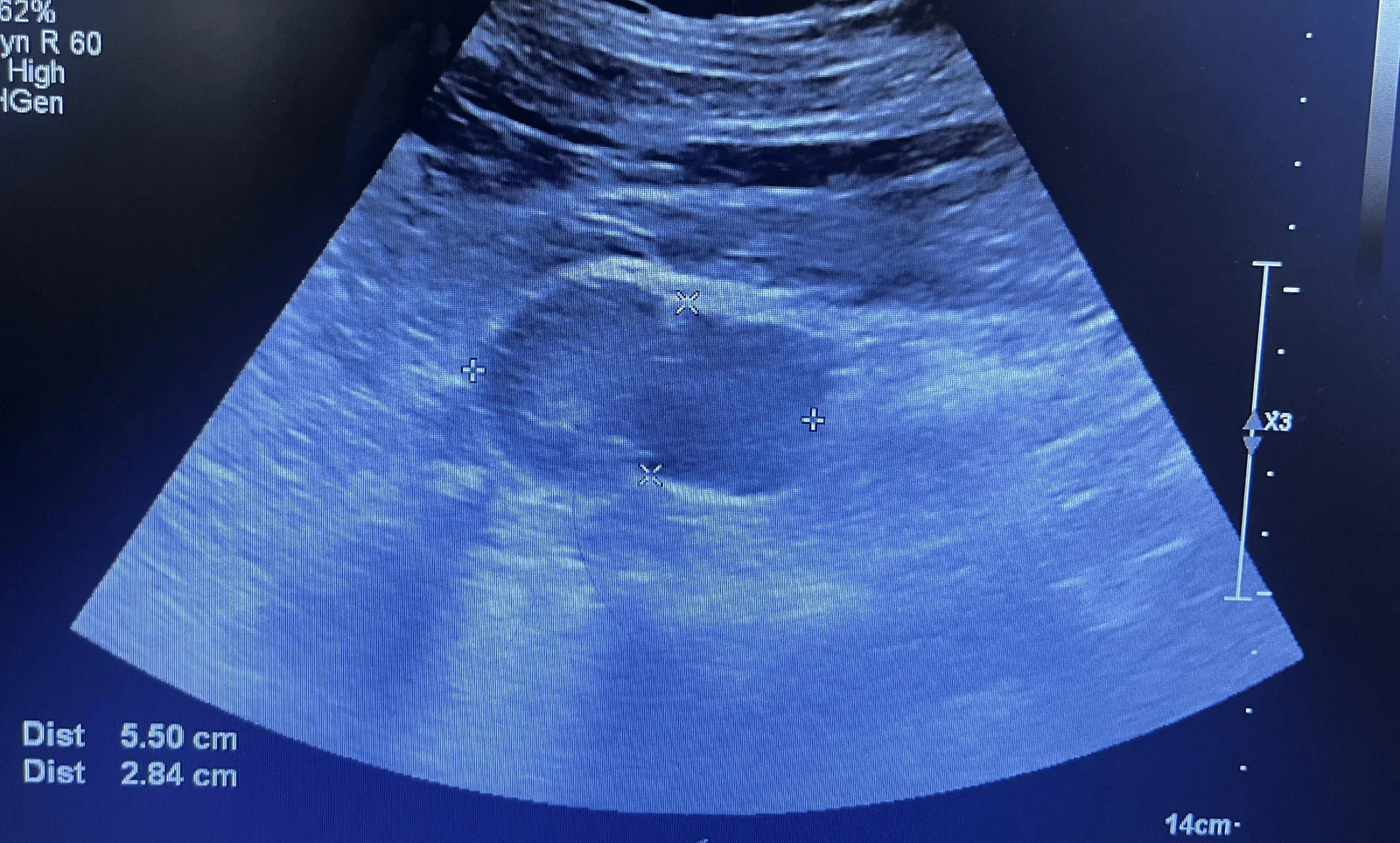
USG abdomen showing a well-defined, lobulated, dumbbell-shaped hypoechoic lesion (4.9 × 3.1 × 3.6 cm) in the right iliopsoas muscle.

Ultrasonography of the abdomen showed a well-defined, lobulated, dumbbell-shaped hypoechoic lesion (4.9 × 3.1 × 3.6 cm) within the right iliopsoas muscle with internal vascularity and cystic areas. The appendix was normal (Figure 1).

**Figure 2:**
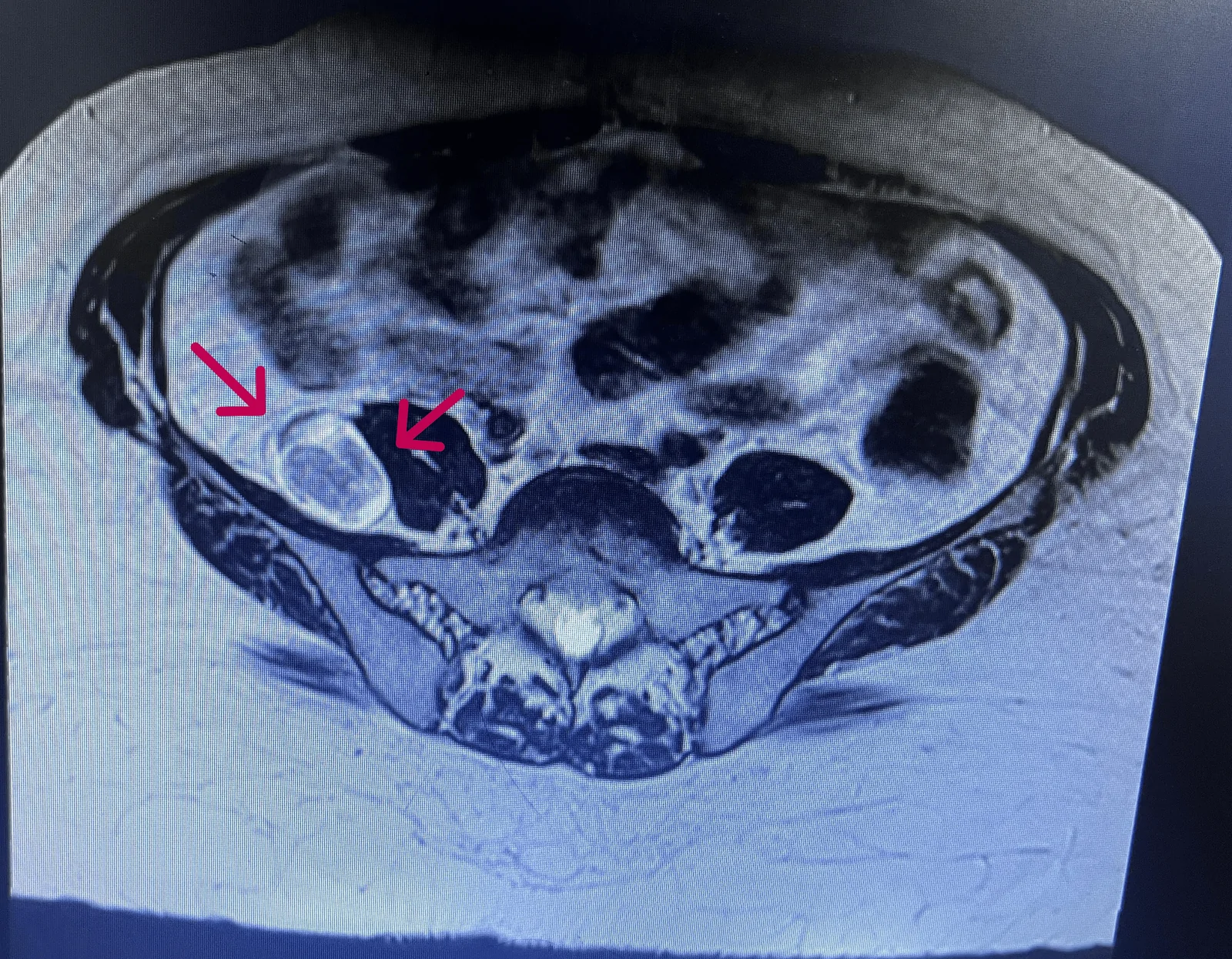
Axial MRI of the pelvis demonstrating a well-defined, dumbbell-shaped retroperitoneal lesion (3.5 × 4.3 × 5.4 cm) (arrows) located along the lateral border of the right psoas muscle at the L4-S1 level. The lesion abuts the right psoas muscle medially and the iliacus muscle posteriorly, with preserved fat planes and no radiological evidence of muscle invasion or adjacent tissue infiltration, findings consistent with a peripheral nerve sheath tumor.

MRI of the pelvis revealed a dumbbell-shaped lesion (3.5 × 4.3 × 5.4 cm) lateral to the right psoas muscle at the L4-S1 level, abutting the psoas medially and iliacus posteriorly without evidence of infiltration--suggestive of a peripheral nerve sheath tumor (Figure 2).

**Figure 3:**
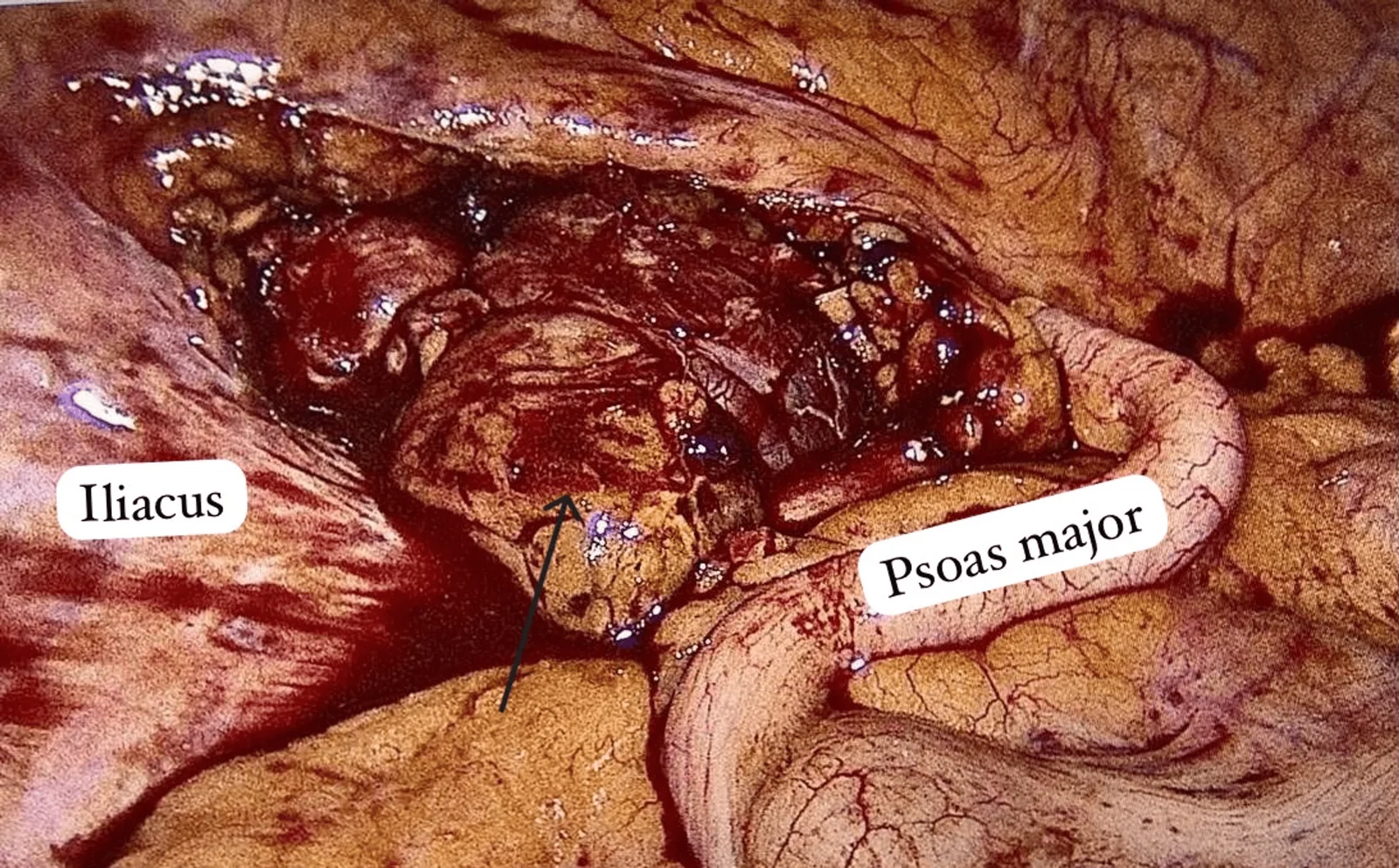
Laparoscopic intraoperative view showing a 5 × 4 cm multilobulated retroperitoneal mass (arrow) situated along the lateral border of the right psoas muscle. The small bowel has been retracted medially to expose the retroperitoneal space, and circumferential dissection is being performed while preserving adjacent neurovascular structures.

After pre-anesthetic evaluation, the patient underwent laparoscopic exploration and excision of the retroperitoneal mass, which revealed a multilobulated lesion (with the largest measuring 5 × 4 cm) along the anterolateral border of the right psoas muscle. Direct continuity between the mass and the LFCN was confirmed intraoperatively by tracing the nerve fascicle into the lesion (Figure 3).

Intraoperative neuromonitoring was done using somatosensory evoked potential (SSEP) of the sensory LFCN, together with monitoring of the adjacent motor muscles (iliacus and psoas), to detect nerve stimulation during dissection. The tumor was dissected off the nerve fascicle using an intracapsular technique, and the LFCN was anatomically preserved throughout. The lesion was excised *in toto*, and the specimen was retrieved through a small suprapubic incision and sent for histopathological examination.

**Figure 4:**
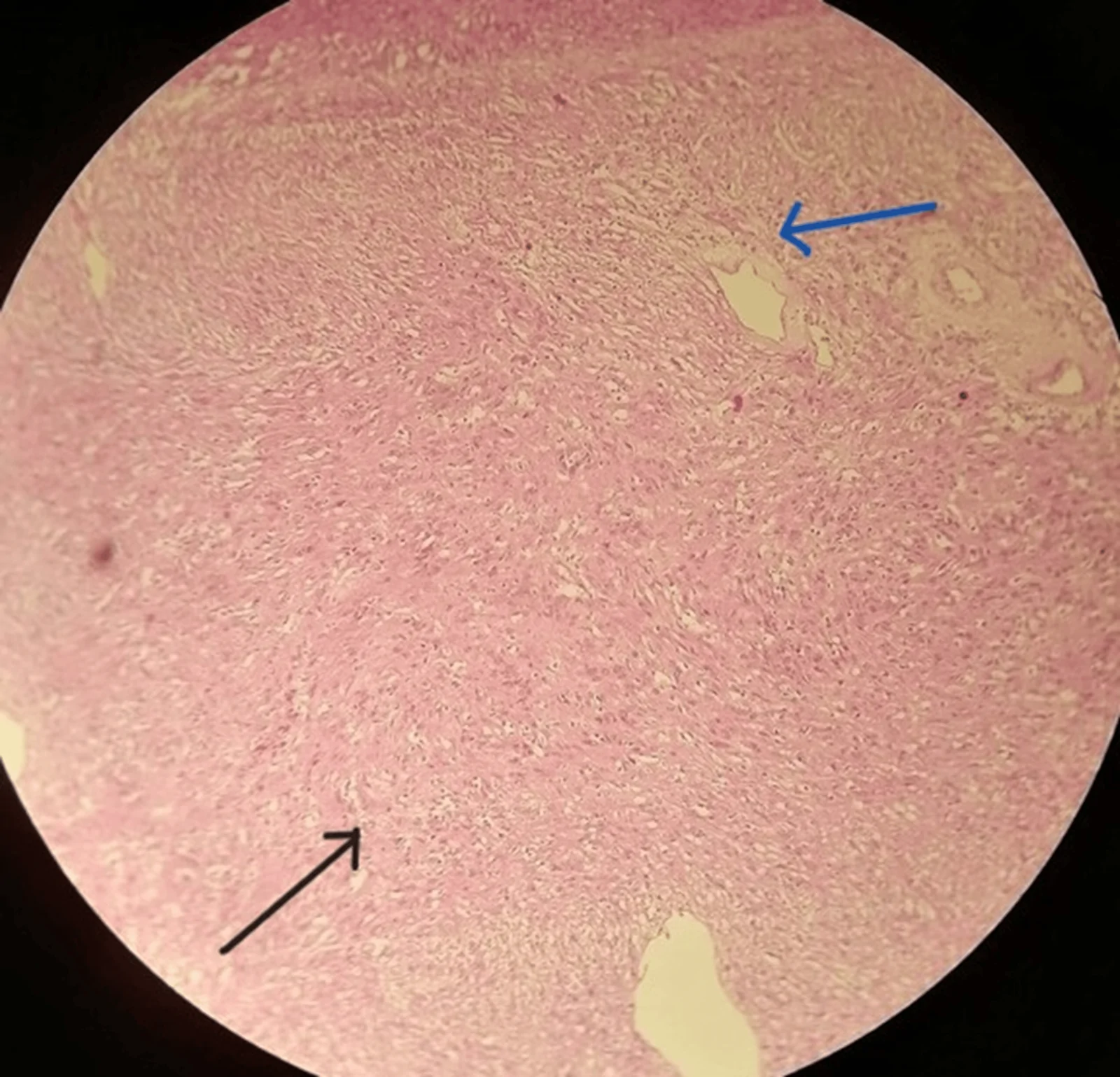
Histopathology of the mass showing alternating hypercellular Antoni A cells (Black arrow) and hypocellular Antoni B cells (Blue arrow) with Verocay bodies and minimal mitotic activity, confirming a benign schwannoma.

Gross examination showed a pale yellow, homogeneous, encapsulated tumor. Microscopy revealed an encapsulated neoplasm with alternating hypercellular (Antoni A) and hypocellular (Antoni B) areas with Verocay bodies and minimal mitotic activity without atypia. Focal cystic degeneration, foamy macrophages, and lymphoid aggregates were noted (Figure 4). With these findings, a final diagnosis of benign schwannoma with degenerative changes was made.

The postoperative period was uneventful, and she was discharged on the third postoperative day with analgesics and antibiotics. She was followed up at one week and 1, 3, and 6 months after surgery, and pain was assessed on a scale of 0-10. The pain score was initially 2/10 at one week. Abdominal pain and cramping leg pain resolved completely at one month. Mild numbness persisted over the anterolateral aspect of the right thigh throughout follow-up, and the burning sensation in both soles also persisted.

## Discussion

LFCN schwannoma as a cause of MP is infrequently reported in the literature. Retroperitoneal schwannomas in general are uncommon and have occasionally been reported to mimic other GI conditions, including a paravertebral schwannoma that presented with symptoms resembling irritable bowel syndrome [6]. While MP is commonly attributed to entrapment at the inguinal ligament, it can also result from tumors of the LFCN itself, which may mimic idiopathic MP and delay diagnosis [2,3]. Makris et al. reported a case of LFCN schwannoma [2]. Compared with this case, our patient’s tumor was larger (5 x 4 cm vs 1.1 cm) and located more proximally, along the psoas muscle at the L4-S1 level, rather than at the inguinal ligament, where LFCN schwannomas are thought to typically arise [2]. This proximal, retroperitoneal location likely explains the right iliac fossa pain, rather than the localized thigh compression symptoms reported in the earlier case. The initial clinical diagnosis, before laboratory investigations and imaging, was chronic appendicitis, as retrocecal appendicitis does not usually present with fever, anorexia, nausea, vomiting, or rebound tenderness, in contrast to classical appendicitis [7].

The surgical approach to the prior case was open decompression through a small inguinal incision, with excision of the tumor and its nerve fascicle, whereas our patient’s tumor required laparoscopic excision with intraoperative SSEP monitoring, given its size, proximity to the psoas muscle, and retroperitoneal location. Both cases achieved symptomatic improvement after excision, though our patient had mild residual thigh numbness at follow-up, whereas the outcome was not specified in comparable detail in the earlier report. The anatomical course of the LFCN, particularly its proximity to the inguinal ligament and psoas muscle, makes it susceptible to compressive lesions, including schwannomas [4,5]. The evidence for MP management remains limited for idiopathic MP [8]. The sensory findings in this patient only partly localize to the LFCN. Loss of pain and temperature sensation over the anterolateral thigh is consistent with LFCN involvement, and the pain reproduced by the LFCN stretch test supports this further. However, the cramping sensation in the right leg, and particularly the bilateral sole burning, falls outside the anatomical territory of an isolated LFCN lesion, which is a purely sensory nerve limited to the anterolateral thigh. As the patient and her family declined further testing, we could not perform nerve conduction studies, which is a limitation of this manuscript. The cramping without numbness could possibly represent referred pain from the primary tumor. Meanwhile, the bilateral burning sensation of the soles, together with its persistence after complete excision of the tumor, argues against a single cause and is more consistent with a coexisting, unrelated process such as a small fiber neuropathy rather than a manifestation of the schwannoma.

In this case, the pelvic compression test did not alleviate symptoms, which is atypical for classical MP [9]. MRI remains the investigation of choice for diagnosing peripheral nerve sheath tumors, as it provides detailed visualization of tumor morphology and its relationship to surrounding structures [10], although imaging alone cannot confirm the nerve of origin; in our patient, this was established only intraoperatively, when direct continuity between the mass and the LFCN fascicle was traced. Ultrasound was a useful first-line investigation, both to characterize the lesion and to confirm a normal appendix. The LFCN stretch test, which reproduced pain along the nerve’s distribution, was a useful bedside maneuver, though it cannot replace imaging [11]. Complete surgical excision with nerve preservation, where feasible, is the treatment of choice for symptomatic LFCN schwannoma and usually results in symptomatic relief [12]. However, as in our patient, residual sensory deficit may persist despite nerve preservation [13]. A complete neurological examination is important in any patient presenting with chronic lower abdominal or thigh pain.

## Conclusions

This case illustrates LFCN schwannoma as a rare structural cause of MP that can mimic chronic appendicitis through the coincidence of right iliac fossa tenderness and a positive psoas sign, and it should be considered in the differential diagnosis of atypical or refractory MP, particularly when conservative management fails. In this patient, surgical excision with nerve preservation resulted in resolution of the abdominal pain and partial improvement in thigh sensory symptoms at six months of follow-up. Bilateral sole burning, which had been present before surgery, persisted unchanged and remained unexplained. This outcome reflects a single case and should not be generalized; in particular, surgical excision of the causative lesion should not be assumed to resolve coexisting sensory findings. A negative pelvic compression test does not exclude tumor-related MP. Symptoms of MP can vary depending on the location of the tumor. As in our case, if the tumor is proximal to the inguinal ligament, the patient can present with abdominal pain. This case underscores the importance of a complete neurological examination in patients with chronic right iliac fossa pain.
